# Ductal Mucus Obstruction and Reduced Fluid Secretion Are Early Defects in Chronic Pancreatitis

**DOI:** 10.3389/fphys.2018.00632

**Published:** 2018-05-29

**Authors:** Anita Balázs, Zsolt Balla, Balázs Kui, József Maléth, Zoltán Rakonczay, Julia Duerr, Zhe Zhou-Suckow, Jolanthe Schatterny, Matthias Sendler, Julia Mayerle, Jens-P. Kühn, László Tiszlavicz, Marcus A. Mall, Peter Hegyi

**Affiliations:** ^1^First Department of Medicine, University of Szeged, Szeged, Hungary; ^2^Department of Translational Pulmonology, Translational Lung Research Center Heidelberg, German Center for Lung Research (DZL), University of Heidelberg, Heidelberg, Germany; ^3^MTA-SZTE Momentum Epithel Cell Signalling and Secretion Research Group, Szeged, Hungary; ^4^Department of Pathophysiology, University of Szeged, Szeged, Hungary; ^5^Department of Pediatric Pulmonology and Immunology, Charité – Universitätsmedizin Berlin, Berlin, Germany; ^6^Department of Internal Medicine A, Universitätsmedizin Greifswald, Greifswald, Germany; ^7^Institute of Radiology, Universitätsmedizin Greifswald, Greifswald, Germany; ^8^Department of Pathology, University of Szeged, Szeged, Hungary; ^9^Berlin Institute of Health, Berlin, Germany; ^10^Institute for Translational Medicine, First Department of Medicine, Medical School, University of Pécs, Pécs, Hungary; ^11^MTA-SZTE Translational Gastroenterology Research Group, Szeged, Hungary

**Keywords:** chronic pancreatitis, mucus, ductal epithelium, experimental pancreatitis, epithelial fluid secretion

## Abstract

**Objective:** Defective mucus production in the pancreas may be an important factor in the initiation and progression of chronic pancreatitis (CP), therefore we aimed to (i) investigate the qualitative and quantitative changes of mucus both in human CP and in an experimental pancreatitis model and (ii) to correlate the mucus phenotype with epithelial ion transport function.

**Design:** Utilizing human tissue samples and a murine model of cerulein induced CP we measured pancreatic ductal mucus content by morphometric analysis and the relative expression of different mucins in health and disease. Pancreatic fluid secretion in CP model was measured *in vivo* by magnetic resonance cholangiopancreatography (MRCP) and *in vitro* on cultured pancreatic ducts. Time-changes of ductal secretory function were correlated to those of the mucin production.

**Results:** We demonstrate increased mucus content in the small pancreatic ducts in CP. Secretory mucins *MUC6* and *MUC5B* were upregulated in human, *Muc6* in mouse CP. *In vivo* and *in vitro* fluid secretion was decreased in cerulein-induced CP. Analysis of time-course changes showed that impaired ductal ion transport is paralleled by increased *Muc6* expression.

**Conclusion:** Mucus accumulation in the small ducts is a combined effect of mucus hypersecretion and epithelial fluid secretion defect, which may lead to ductal obstruction. These results suggest that imbalance of mucus homeostasis may have an important role in the early-phase development of CP, which may have novel diagnostic and therapeutic implications.

## Introduction

Chronic pancreatitis (CP) is a self-sustaining, progressive inflammatory disease, associated with an increased risk for developing pancreatic cancer, impaired quality of life, and markedly decreased life expectancy (Kleeff et al., 2017). The heavy disease burden is still aggravated by the lack of specific therapy, which is due to our poor understanding of the pathomechanism, with special regard to early-phase CP. Obstruction of the pancreatic ducts by mucoprotein plugs and calcified stones are hallmark histological features of CP and it has been postulated that ductal obstruction may be an early event in the pathogenesis. In the 1960s, Sarles et al. (1965) documented the formation of a mucoprotein gel in the small pancreatic ducts of CP patients with apparent focal fibrotic lesions, acinar atrophy, and ductal dilatations upstream to the obstruction, suggesting that ductal plugging occurs prior to parenchymal damage. These features are reminiscent of the ones seen in the pancreas of patients with cystic fibrosis (CF), indicating that impaired cAMP-dependent chloride and bicarbonate secretion by the CF transmembrane conductance regulator (CFTR) anion channel is a key mechanism in the development of ductal plugs. Indeed, diminished ${\text{HCO}}_{3}^{-}$ secretion is a well-known and early defect in CP (Braganza and Rao, 1978; Freedman, 1998), and numerous studies demonstrated that pancreatitis-associated toxic factors, such as ethanol (Maléth et al., 2015), cigarette smoke extract (Raju et al., 2013), bile acids (Venglovecz et al., 2008), and trypsin (Pallagi et al., 2011) display inhibitory effect on bicarbonate secretion by decreasing the activity and the expression of CFTR. Insufficient ion and fluid secretion leads to protein hyperconcentration that predisposes to protein precipitation in the pancreatic juice, which has been demonstrated in alcoholic CP and CF patients (Sahel and Sarles, 1979; Kopelman et al., 1985; Freedman et al., 1993). Another important but often overlooked determinant of pancreatic ductal plugging is mucus production. Ductal cells express mucins, heavily *O*-glycosylated, membrane-bound, or secreted (gel-forming) glycoproteins that generate a protective layer of mucus on the epithelial surface. In many epithelial organs, mucus layer serves as a physical, chemical, and biological barrier with important host-defense functions, while in the pancreas it could provide protection against autodigestion or bile acid reflux. The most frequent pancreatic epithelial proliferative change is mucinous cell hypertrophy, also known as ductal non-papillary hyperplasia, goblet cell metaplasia, or as most recently classified, PanIN-1A (Hruban et al., 2001). It designates the replacement of the normal ductal epithelium by tall columnar cells with basal nuclei and abundant supranuclear mucus that may occur in ducts of every size. Although there are various reports on the natural prevalence of mucinous cell hypertrophy (Walters, 1965; Cubilla and Fitzgerald, 1976; Allen-Mersh, 1988; Sipos et al., 2009), a comparative study found higher frequency in CP patients, than in controls without pancreatic disease (60% and 16%, respectively) (Andea et al., 2003), suggestive of increased mucus secretion. Supporting this notion, there are reports of increased mucopolysaccharide concentration (Wakabayashi and Takeda, 1976) and abundant mucus content of precipitated plugs in the pancreatic juice of CP patients (Harada et al., 1981). However, several studies led to the general belief that protein precipitates originate solely from acinar cell secretion (Guy et al., 1983; Freedman et al., 1993; Ko et al., 2012), which could be due to a number of challenges of mucus biochemistry, as isolating, detecting, and visualizing large molecular weight and highly glycosylated mucins require specific methodology (Mucins, 2018).

The biophysical properties of mucus are essentially determined by ${\text{HCO}}_{3}^{-}$ (Quinton, 2008). In mucus-producing cells, negatively charged mucin macromolecules are highly condensed in secretory granules, where high Ca^2+^ concentration and low pH (∼6) provide cationic shielding of intragranular charge. Upon exocytosis mucin polymers rapidly expand their volume up to 1000-fold and ${\text{HCO}}_{3}^{-}$ promotes this swelling and hydration process by chelating Ca^2+^ ions and buffering protons, thereby facilitates the formation of a proper gel (Chen et al., 2010). In CF-affected organs, the lack of CFTR-mediated ${\text{HCO}}_{3}^{-}$ secretion leads to a tenacious, viscous mucus, and importantly, it has been shown that adding bicarbonate can restore the rheological properties of CF mucus (Gustafsson et al., 2012). It is plausible that increased mucin secretion together with reduced ductal bicarbonate/fluid transport in CP generates a thick, viscous mucus that may obstruct the outflow of pancreatic enzymes. Defective mucus hydration, as a result of a primary defect in epithelial ion transport or due to hypersecretion of “dry” mucin macromolecules, has been established as a key pathomechanism in a spectrum of muco-obstructive lung diseases, such as CF, chronic obstructive pulmonary disease (COPD), and asthma (Button et al., 2016). To date, a number of drugs are available that target mucus dehydration in these respiratory disorders and there are ongoing efforts to develop new strategies for more effective treatment options (Randell and Boucher, 2006; Balsamo et al., 2010; Tildy and Rogers, 2015; Mall, 2016). Despite these advances, our current understanding of the role of mucus obstruction in the pancreas remains limited. In the present study, we aimed to investigate the early phase of CP. Utilizing human tissue samples and a murine model of CP we have measured the pancreatic ductal mucus content and the relative expression of different mucins in health and disease. Using *in vivo* and *in vitro* techniques, we have investigated pancreatic fluid secretion over the course of experimental CP and we correlated these with the time-changes of mucin production. Finally, to evaluate possible effect of CFTR we analyzed mucin secretion changes in *Cftr* knock-out mice pancreata.

## Materials and Methods

### Ethical Approval

All experiments were approved by local ethical committees on investigations involving animals at University of Szeged, University of Heidelberg, and at University of Greifswald. Studies involving human tissues were approved by the Human Investigation Review Board at University of Szeged.

### Human Pancreas Tissues

Formalin-fixed paraffin-embedded human CP tissue samples were provided by the Department of Pathology, University of Szeged. Control tissue samples were obtained from brain dead organ donors in cooperation with the Hungarian Organ Coordination Office.

### Experimental Animals

Experimental CP was modeled in 8- to 20-week-old mice on FVB/N background. Chronic pancreatic injury was induced by repetitive administration of cerulein with a treatment protocol as follows (Xue et al., 2015): 6-times hourly intraperitoneal injections of 50 μg/kg cerulein, 3 days a week, during a 1–2–3–4-week-period. Controls were treated with physiological saline. Two days after the last injection mice were sacrificed and pancreata were removed. Gut-corrected CF (*Cftr^-/-^*) mice overexpressing human CFTR in the intestine under control of the fatty acid-binding protein promoter (*Fabp-hCFTR-Cftr^-/-^*) on the FVB/N background were kindly provided by Dr. Jeffrey A. Whitsett and genotyped, as previously described (Zhou et al., 1994).

### Histology and Mucus Morphometry

Pancreata were immersion fixed in 4% buffered formalin and embedded in paraffin. To detect mucus, 5 μm sections were cut and stained with alcian blue periodic acid-Schiff (AB-PAS) staining. Images were taken with an Olympus IX-71 microscope interfaced with a SIS Colorview I Camera Set (Olympus, Hamburg, Germany) using the 10× and 40× objective. To quantify secreted mucus, morphometric analysis of stained sections were carried out by determining mucus volume density using CellF Software, as previously described (Mall et al., 2008). In brief, the length of the basal membrane of the ductal epithelium was measured by the interactive image measurement tool, and the AB-PAS positive surface area within this boundary was measured by phase analysis according to the automatic threshold settings of the software. The volume density of ductal epithelial mucus, representing the volume of ductal mucus content per surface area of the mucus basal membrane (nl/mm^2^), was determined from the epithelial surface area of AB-PAS positive mucus and the length of the basal membrane of the ductal epithelium. Luminal mucus content relative to luminal area was determined in a similar fashion. In case of mixed (pink proteinaceous and alcian-blue positive mucus) intraluminal material an additional phase analysis step was introduced to determine the overall area of luminal content within the lumen (Supplementary Figure 1). In case of human samples, one section per tissue block was stained and all ducts within the given section were analyzed. To determine the sampling protocol in mice, we examined homogeneity of mucus changes within the tissue by preparing serial sections of the pancreas of a 4-week cerulein-treated animal, where we measured comparable mucus volume densities throughout the pancreas (reviewed but not shown). Therefore, we sampled one section per tissue and all ducts were analyzed within 10 random fields of view with a 10× objective.

### Real Time RT-PCR

We have analyzed expression of different mucins in human and mouse pancreata. Total RNA was isolated from frozen tissue using RNeasy Mini Kit (Qiagen, Hilden, Germany), while RNA from paraffin-embedded tissue was isolated with RNeasy FFPE Kit (Qiagen, Hilden, Germany). Two micrograms of RNA was reverse transcribed using High-Capacity cDNA Reverse Transcription Kit (Applied Biosystems, Foster City, CA, United States) in the presence of RNase inhibitor RNasin Plus (Promega, Fitchburg, WI, United States). Real-time PCR reactions of mouse samples were performed with Taqman RT-PCR assays (MM00449604_m1 *Muc1*, MM00466886_m1 *Muc4*, MM00466391_m1 *Muc5b*, MM00725165_m1 *Muc6*; Thermo Scientific, Darmstadt, Germany), while analysis in human samples was performed with maxima SYBR Green/ROX qPCR Master Mix (2×) (Fermentas, St. Leon-Rot, Germany). Human primers were designed according to the reference sequences of *MUC1*, *MUC4*, *MUC5B*, *MUC5AC*, and *MUC6* genes (**Table 1**). Reactions were carried out with ABI PRISM 7000 Sequence Detection System (Applied Biosystems, Foster City, CA, United States) platform with the following conditions: 10 min initial denaturation at 95°C, followed by 40 two-step cycles: 15 s at 95°C and 1 min at 60°C. Threshold cycle (CT) values were determined using the 7000 Sequence Detection System Software V.1.2.3. Relative expression was calculated using the comparative CT method (ΔΔCT method). Expression level of the gene of interest was first normalized to the human glyceraldehyde-3-phosphate dehydrogenase (*GAPDH*) or mouse β-actin (*Actb*) internal control gene (ΔCT) and then to expression levels measured in control tissue (ΔΔCT). Results were expressed as fold changes calculated with the formula 2^-ΔΔCT^.

**Table 1:** Primer sequences used for human mucin transcript analysis (5’→3’).

*MUC1-Forward*	AGACGTCAGCGTGAGTGATG
*MUC1-Reverse*	AGAACACAGACCAGCACCAG
*MUC4-Forward*	CTCAATCAGTACCCGCCCTC
*MUC4-Reverse*	GCTGTCTCTGAGCGTGAAGT
*MUC5AC-Forward*	TCCACTATGAGTGCGAGTGC
*MUC5AC-Reverse*	AGGACATAGGTGCAGTTGCC
*MUC5b-Forward*	TATGTGCTGACCAAGCCCTG
*MUC5b-Reverse*	GCTCAGTGTCACGCTCTTCA
*MUC6-Forward*	CAGTACACACAGGAGGCCAA
*MUC6-Reverse*	GGAGCAGTTGTAGCAGCCTT
*GAPDH-Forward*	CACCATCTTCCAGGAGCGAG
*GAPDH-Reverse*	GACTCCACGACGTACTCAGC

### Magnetic Resonance Imaging of Pancreatic Fluid Secretion *In Vivo*

Magnetic resonance imaging (MRI) was performed to measure the total excreted volume of pancreatic fluid after secretin stimulation in control and 4-week cerulein-treated mice (Maléth et al., 2015). For this purpose, animals were allowed free access to pineapple juice for 12 h before the MRI examination, serving as an oral negative contrast agent. Small animal MRI was performed in a 7.1-Tesla animal scanner (Bruker, Ettlingen, Germany) using a mouse whole body coil. Strong T2-weighted series of the entire abdomen were acquired before and after retroorbital injection of 10 IU units/kg secretin (ChiroStim, ChiRhoClin, Burtonsville, MD, United States). Both MRI sequences (before and after secretin injection) were acquired using the same image protocol with the following image parameters: TR/TE 4400/83 ms; flip angle: 180°; matrix 256 × 256; field of view 40 × 40 mm; bandwidth 315 hz/pixel; slice thickness 1 mm; 20 slices. One observer with >12 years of experience in GI radiology reviewed the images using the software Osirix (version 5; Pixameo, Bernex, Switzerland). First, we reduced image noise to minimize artifacts in images. Second, fluid excretion into the small intestine was segmented in each slice. Fluid excretion was defined as areas in the bowel with high signal intensity. Care was taken to avoid artifacts caused by magnetic inhomogeneity and motion especially bowel motion. The volume of intestinal fluid was assessed before and after secretin stimulation. From these data total excreted volume was assessed. Measured values were normalized to control mice.

### Measurement of Pancreatic Fluid Secretion *In Vitro*

Fluid secretion into the closed luminal space of the cultured mouse pancreatic ducts was analyzed using a swelling method developed by Fernández-Salazar et al. (2004). Briefly, intra/interlobular pancreatic duct fragments were isolated by microdissection from the pancreas of control and cerulein-treated mice. After 6 h of incubation sealed ducts were attached to a coverslip precoated with poly-L-lysine in the base of a perfusion chamber (0.45 ml). Bright-field images were acquired at 1 min intervals using a CCD camera (CFW 1308C, Scion Corporation, Frederick, MD, United States). First, baseline images were taken in HEPES-buffered solution (140 mM NaCl, 5 mM KCl, 1 mM CaCl_2_, 1 mM MgCl_2_, 10 mM Glucose, 10 mM HEPES, pH 7.5), then chamber was perfused with ${\text{HCO}}_{3}^{-}$ containing solution (115 mM NaCl, 5 mM KCl, 25 mM NaHCO_3_, 1 mM CaCl_2_, 1 mM MgCl_2_, 10 mM Glucose). To stimulate ductal secretion 100 μM 3-isobutyl-1-methylxanthine (IBMX) and 5 μM forskolin were added to the perfusate. The integrity of the duct wall was checked at the end of each experiment by perfusing the chamber with a hypotonic solution (HEPES-buffered solution diluted 1:1 with distilled water). Digital images of the ducts were analyzed using ImageJ software to obtain values for the area corresponding to the luminal space in each image.

### Statistics

Quantitative variables were described as mean ± SE. Significant differences between groups were determined by Student’s *t*-test or by analysis of variance with Dunnett’s *post hoc* analysis. All the analyses were performed with GraphPad Prism (San Diego, CA, United States).

## Results

### Mucus Content Is Increased in Small Ducts in Chronic Pancreatitis

**FIGURE 1:**
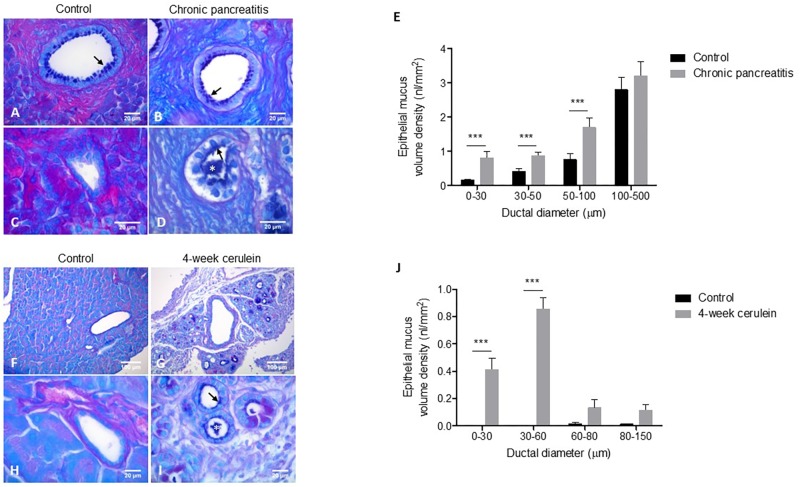
Representative morphology of AB-PAS stained human pancreata **(A–D)**. The epithelial cells of an interlobular duct in control **(A)** and CP **(B)** tissue are lined with a thin layer of mucus (arrows). A small intralobular duct in control tissue **(C)** shows no mucus staining. An intralobular duct in CP **(D)** is filled with inspissated mucus (asterisk). Morphometric analysis of epithelial mucus volume density of pancreatic ducts in control and CP human tissues **(E)**. *N* = 9–9 tissue samples, *N* = 178–185 total number of analyzed ducts in control and CP, respectively, *N* = 15–71 ducts per group. Data are shown as pooled average over all ducts in control and CP group. Representative morphology of AB-PAS stained mouse pancreata **(F–I)**. A large interlobular duct in control tissue shows no mucus staining **(F)**. A large interlobular duct in 4-week cerulein induced CP **(G)** with little mucus staining surrounded by numerous small ducts that contain mucus. A small intralobular duct in control tissue with no mucus **(H)**. A cluster of small ducts in CP shows epithelial mucus lining (arrows) and inspissated mucus (asterisk) **(I)**. Morphometric analysis of epithelial mucus volume density of pancreatic ducts in control and 4-week cerulein-treated mice **(J)**. *N* = 9–9 tissue samples and *N* = 64–186 total number of analyzed ducts in control and 4-week cerulein treated mice, respectively, *N* = 4–116 ducts per group. Data are shown as pooled average of all ducts in control and treated group. ^∗∗∗^*P* < 0.001.

Utilizing AB-PAS for staining mucus and quantitative histological approach we compared the pancreatic morphology of human control and CP tissue samples. AB-PAS-stained sections revealed a predominantly alcian-blue positive staining of the pancreatic mucus, indicating the presence of acidic mucopolysaccharides. Mucus was localized to the epithelial surfaces and to the luminal area of pancreatic ducts. In the case of control pancreata, there was little or no mucus present in intralobular ducts, while in CP tissue small ducts exhibited substantial amounts of mucus that often completely plugged the lumen (**Figures 1A–D**). Morphometric analysis revealed a significant increase of epithelial mucus content in small ducts (diameter <100 μm), while no difference was observed in large interlobular ducts (**Figure 1E**). In concordance with the epithelial mucus measurements we found increased intraluminal mucus in the smallest ducts in CP, while proteinaceous material was less prevalent, which could reflect decreased acinar secretion, i.e., exocrine dysfunction in CP (Supplementary Figures 2A,B).

Next, we examined the mucus changes in experimental CP. Pancreatic damage was induced with 4 weeks of cerulein injections in mice. In contrast to the human situation, alcian-blue positive staining was virtually absent in the ducts of control mice. Although intestinal-type goblet cells were present at the level of the common bile duct, they shortly disappeared from the main duct (data not shown). In cerulein-induced CP tissue formation of novel ductal structures due to acinar-to-ductal metaplasia (ADM) was apparent. Similarly to human CP, we found alcian-blue positive mucus on the surface of the ductal epithelium and intraluminally in small ducts (**Figures 1F–I**). Morphometric analysis showed that mucus volume density was significantly higher in small ducts (diameter <60 μm) in cerulein-treated mice (**Figure 1J**). Quantification of the luminal compartment revealed increased amount of intraluminal mucus in the smallest ducts as well (Supplementary Figure 2C).

### Secretory Mucins Are Differentially Expressed in Human and Experimental Chronic Pancreatitis

**FIGURE 2:**
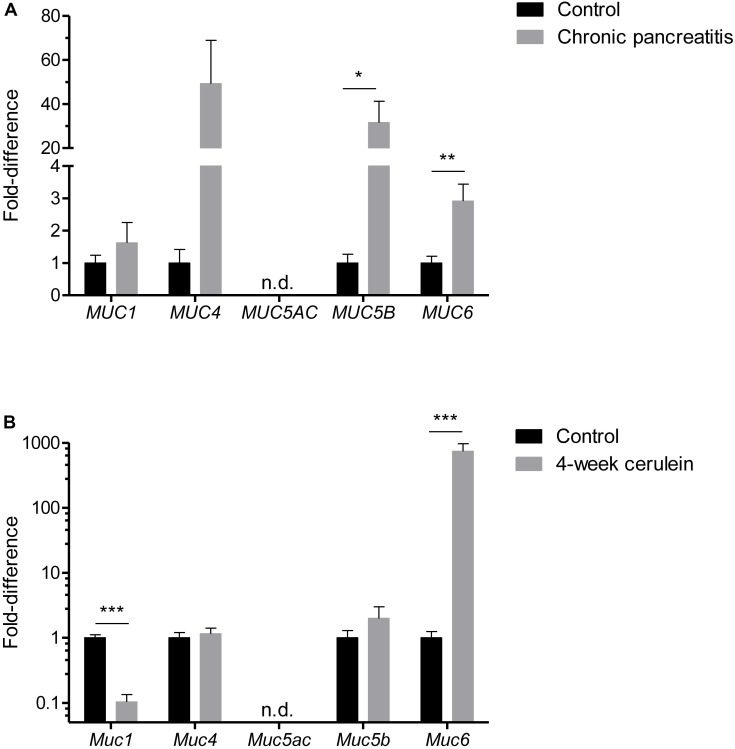
Mucin mRNA expression panel in human **(A)** control and CP pancreatic tissue samples. *N* = 9–11. Mucin mRNA expression panel in mouse **(B)** control and 4-week cerulein-treated tissue samples. *N* = 9–9. n.d. = not detected. ^∗∗∗^*P* < 0.001, ^∗∗^*P* < 0.01, and ^∗^*P* < 0.05.

Mucus accumulation in the small ducts could be a result of hypersecretion or due to insufficient removal/flush-out. To identify secretory changes, we analyzed tissue mRNA expression of different mucins in human. We found that secreted mucins *MUC5B* and *MUC6* were significantly upregulated in CP (**Figure 2A**) suggesting epithelial mucus hypersecretion. Another secreted mucin *MUC5AC* was not detectable in most of the samples (8/9 control and 10/11 CP). Membrane-bound mucins *MUC1* and *MUC4* were not significantly altered, although normalization of MUC4 samples was hampered as many samples were below detection limit (6/9 control and 4/11 CP).

In cerulein-induced murine CP we found that *Muc6*, but not *Muc5b* was upregulated by three orders of magnitude, indicating that *Muc6* glycoprotein is the dominant secreted mucin in the mouse pancreatic duct (**Figure 2B**). Membrane-bound mucin *Muc1* was downregulated that could be attributed to lower number of acinar cells, that also express *Muc1*.

### Pancreatic Ductal Fluid Secretion Is Decreased in Experimental CP *In Vitro* and *In Vivo*

**FIGURE 3:**
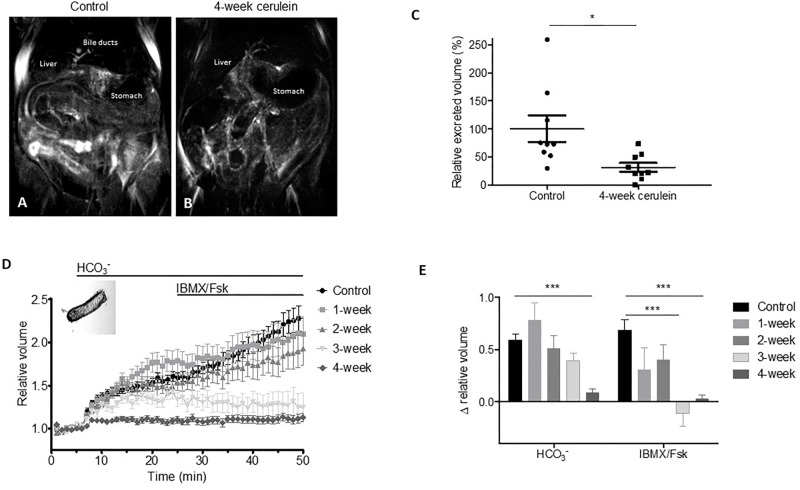
Representative images of magnetic resonance imaging cholangiopancreatography (MRCP) upon secretin stimulation in saline-treated control and 4-week cerulein-treated mice **(A,B)**. Relative excreted volume measured by MRCP in control and 4-week cerulein-treated mice **(C)**. *N* = 9–9 mice per group. Data obtained from two independent repeats were normalized to the respective controls at end-point. Optical measurement tracings **(D)** and data summary **(E)** of pancreatic ductal swelling *in vitro* of isolated duct fragments in saline-treated control and 1–2–3–4-week cerulein-treated mice. Bar charts summarize the relative changes of volume in ${\text{HCO}}_{3}^{-}$ containing buffer (between 5 and 25 min) and upon IBMX/forskolin stimulated secretion (between 25 and 50 min). *N* = 5–14 mice per group, *N* = 22–84 isolated ducts per group. Data are shown as pooled average of all ducts in treatment groups. ^∗∗∗^*P* < 0.001.

Defective mucus hydration may develop if increased mucus secretion is accompanied by an epithelial ion and fluid transport defect. To assess the pancreatic ductal function in CP we applied two different techniques. First, we used magnetic resonance imaging cholangiopancreatography (MRCP) to measure total excreted volume in control and 4-week cerulein-treated mice *in vivo* (**Figures 3A,B**). Two days after the last injection, MRCP images were taken on baseline and upon secretin stimulation. We found that the total excreted volume was significantly decreased in CP mice compared to the controls (**Figure 3C**). In order to gain insight into the time-changes of ion and fluid transport, we examined pancreatic ductal secretory function *in vitro* using different cerulein treatment time-series. We isolated pancreatic duct fragments from control and 1–2–3–4-week cerulein-treated mouse pancreata, and measured fluid secretion by the swelling assay. Basal ductal secretion in ${\text{HCO}}_{3}^{-}$ containing buffer in 4-week cerulein-treated mice was significantly lower, than in controls. Upon administration of IBMX and forskolin, stimulated fluid secretion was markedly reduced in 3- and 4-week cerulein-treated mice (**Figures 3D,E**).

### Muc6 Is Associated With Altered Ion Transport

**FIGURE 4:**
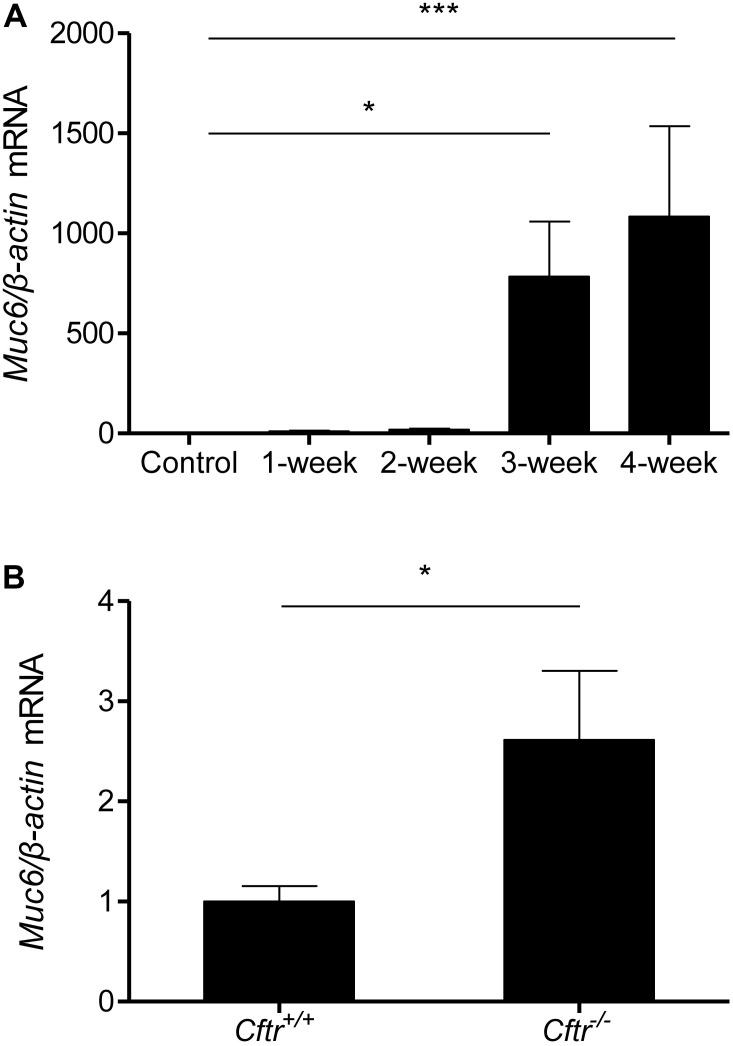
Muc6 mRNA expression analysis in pancreatic tissue from control and 1–2–3–4-week cerulein-treated mice **(A)**. *N* = 9–11, ^∗∗∗^*P* < 0.001, ^∗^*P* < 0.05. Muc6 mRNA expression analysis in pancreatic tissue from 29- to 31-week-old *Cftr* knock-out mice and wild-type littermates **(B)**. *N* = 5–6, ^∗^*P* < 0.05.

Next, we aimed to determine the relationship of the development of the mucus obstruction phenotype and impairment of ductal fluid secretion over time. We examined *Muc6* transcript levels in pancreata from 1–2–3–4-week cerulein-treated mice and found significant upregulation at 3 and 4 weeks of treatment (**Figure 4A**), parallel to the timing of changes of fluid secretion in CP. To further investigate the relationship between *Muc6* and altered epithelial ion transport we examined *Muc6* mRNA expression in the pancreas of long-lived (29–31 weeks old) *Cftr^-/-^* mice in comparison with their age-matched *Cftr^+/+^* littermates. *Muc6* mRNA was approximately threefold upregulated in *Cftr^-/-^* pancreas (**Figure 4B**).

## Discussion

In our study we demonstrate increased epithelial mucus production in the small pancreatic ducts in the early phase of CP, which is paralleled by an impairment of pancreatic ductal fluid secretion. The evidence we present here supports that mucus dehydration is an important aspect of the early-pathogenesis and argues the rationale for therapeutic targeting of defective mucus in CP.

Inspissation of pancreatic ducts with mucoprotein material has been long observed in CP, while pathology studies suggested that mucinous cell hypertrophy and differential expression of mucins associate with the disease (Hruban et al., 2008); however, there has been no detailed quantitative and qualitative characterization of mucus produced by the pancreatic ducts in health and CP. In this study, we show higher amounts of mucus in the epithelial cells and also in the lumens of small pancreatic ducts in human CP, which was also replicated in a murine model of cerulein-induced CP. In the normal pancreas, ductal cells secrete high amounts of alkaline fluid that flushes out acidic, protein-rich acinar secretion. Intercalated and intralobular ducts highly express apical CFTR and they are the preferential sites of ${\text{HCO}}_{3}^{-}$ secretion (Ishiguro et al., 2012), while in acute and CP CFTR expression decreases in these cells (Maléth et al., 2015). The finding that the mucus phenotype localizes to the small peripheral ducts suggests a common dysregulation with ion transport in CP.

Consistent with previous reports (Andrianifahanana et al., 2006; Nagata et al., 2007) we found that *MUC5B* and *MUC6* secretory mucins are upregulated in human CP, indicating a hypersecretion of mucus. *MUC5B* is also expressed in the airways, salivary glands, endocervix, and gall bladder, while *MUC6* in the stomach, duodenum, gall bladder, and the seminal vesicle (Bartman et al., 1998). Interestingly, *MUC6* transcript was found in the centroacinar cells and intralobular ducts in the fetal and postnatal pancreas with an expression pattern similar to CFTR. Moreover, the same study showed MUC6 glycoprotein in the luminal material in the pancreas of CF patients (Reid et al., 1997). Another report also suggested increased Muc6 levels in *Cftr*^-^*^/^*^-^ mouse pancreata (Gouyer et al., 2010, p. 6), supporting the connection of MUC6 with ductal secretory dysfunction. Strikingly, in cerulein-induced experimental CP model *Muc6* solely was greatly overexpressed, which implies that the histologically observed mucus increase is a result of selective Muc6 mucin hypersecretion.

These data raise the typical “chicken or the egg” question whether increased mucus in the small ducts in CP is a result of mucus hypersecretion complicated by a defective flush-out mechanism, or primarily of an impaired ion and fluid transport that leads to accumulation and obstruction of constitutively secreted mucins, while mucus hypersecretion being secondary to inflammatory stimulus. To investigate ductal secretory function, we utilized two complimentary methods, MRCP *in vivo* and ductal swelling assay *in vitro* in murine CP. The cerulein-induced CP model causes repetitive injuries in the pancreas that mirror recurrent acute attacks developing into CP as it occurs in some patients (Lara and Levy, 2004). Although supramaximal cerulein stimulation primarily affects acinar cells, resulting in vacuolization, diminished secretion, and intrapancreatic enzyme activation, the importance of ductal function in this model has been outlined by several studies. In cerulein-induced acute pancreatitis, fluid hypersecretion was observed in rats (Czakó et al., 1997); furthermore, genetic deletion of *Cftr* (Dimagno et al., 2005) as well as deletion of its trafficking regulator Na^+^/H^+^ exchanger regulatory factor-1 (*Nherf-1*) (Pallagi et al., 2014) resulted in a more severe acute pancreatitis phenotype in mice. These studies indicate a protective wash-out mechanism by the ducts relevant to this model, and to our knowledge, we demonstrate for the first time evidence of an impaired ductal function in cerulein-CP.

To gain insight into the development of the secretion defect, we performed *in vitro* time-series experiments with different treatment regimens and found that ductal fluid secretion begins to decrease after 3 weeks of cerulein treatment. More specifically, the cAMP-stimulated secretion abolished, which could indicate CFTR dysfunction, although the authors admit that time-matched traces without IBMX/Forskolin stimulation or application of specific CFTR inhibitors would allow more appropriate determination of CFTR function and the lack thereof. After 4 weeks of treatment we observed a decrease of basal secretion as well, where one could speculate further involvement of anion exchangers.

To correlate these time-changes with the development of mucus phenotype, we examined Muc6 expression in different treatment schedules. Marked overexpression was observed after 3 weeks treatment, which corresponded to the time-point of deteriorating ductal secretion, showing that mucus hypersecretion is paralleled by an epithelial electrolyte transport defect. As a proof of concept, we examined Muc6 expression in long-lived *Cftr^-/-^* mice. These mice do not exhibit overt pancreas pathology, but age-dependently they develop mild features consistent with progressive ductal obstruction, such as lower acinar cell count and distended ductal lumina with inspissated material (Durie et al., 2004). Although we did not see mucus histologically (data not shown) *Muc6* transcript analysis showed an overexpression in *Cftr^-/-^* mice, demonstrating that Muc6 can be induced by a basic transport defect.

Taken together these data could suggest a common dysregulation of CFTR and mucus, namely: (i) mucus accumulates in the small ducts, where CFTR is localized, (ii) cAMP-dependent secretion defect is paralleled by mucus hypersecretion, and (iii) *Muc6* is overexpressed in *Cftr^-/-^* mouse pancreata. These observations would also argue that increased Muc6 secretion in CP develops early with impaired ion transport, rather than as a consequence of sustained inflammation. This possibility would be analogous to the pathomechanism of mucus stasis in smoking-related COPD. Cigarette smoke exposure induces mucin overexpression and impairs CFTR-mediated Cl^-^ secretion in the airways, which results in decreased mucociliary clearance and mucus plugging (Button et al., 2016). In contrast to tobacco-induced acquired CFTR deficiency, mucus hypersecretion in CF lung disease develops secondary to inflammatory response. In CF airways, the underlying transport defect causes airway surface dehydration, mucus hyperconcentration, and impaired mucus clearance, which triggers chronic inflammation and microbial infections. In this inflammatory environment, numerous cytokines, proteases, and bacterial products mediate mucus hypersecretion and goblet cell metaplasia, further aggravating mucus plugging (Button et al., 2016).

Alternatively, increased mucus production may develop as a response to inflammation in pancreatitis, preceding ductal obstruction. In cerulein, hyperstimulation, inflammation, and cell death appear already 4–6 h after injury of and take 12–24 h to reach maximum levels (Lerch and Gorelick, 2013). During the course of cerulein CP oxidative stress, inflammatory cytokine production and neutrophilic infiltration are present already after the first attack and it is plausible that these could be potent signals for increased mucus secretion in ductal cells. Due to cellular injury in murine CP ADM develops through dedifferentiation of acinar cells (Storz, 2017). In ADM a developmental program is activated with the expression of genes that are active during embryonic development or normally restricted to uninjured ductal cells and is believed to represent a defense mechanism in pancreatitis. Increased mucus secretion could also be part of this repair program possibly providing a barrier in leaky ducts with increased paracellular permeability.

However, imbalance between mucus production and ductal flush-out mechanism may be detrimental which leads to mucus plugging, delaying the outflow of digestive enzymes, and triggering intrapancreatic zymogen activation. Our data indicate defective mucus hydration and increased mucus viscosity in the small pancreatic ducts due to mucus hypersecretion and impaired ductal fluid secretion. The luminal content we observed histologically predominantly consisted of mucus in the small ducts in CP (Supplementary Figures 2A,B). This mucoprotein material, a viscous mixture of hyperconcentrated mucus and acinar secretion, can readily block narrow lumens, or may also serve as a nidus for calcification. There is a body of evidence on the importance of ductal wash-out mechanism on the integrated function of acinar and ductal cells (Hegyi and Petersen, 2013). Impaired epithelial ion and fluid secretion due to CFTR dysfunction leads to acidification of intraluminal pH (Takács et al., 2013), which may lead to premature intra-acinar and intraluminal trypsinogen activation (Sherwood et al., 2007; Pallagi et al., 2011), while active trypsin can further inhibit CFTR via luminal PAR_2_ receptor cleavage (Pallagi et al., 2011). Moreover, elevated upstream pressure in plugged ducts can further decrease ${\text{HCO}}_{3}^{-}$ secretion via neuroendocrine signaling (Suzuki et al., 2001). Altogether, ductal obstruction generates a vicious cycle of pathological processes that may explain the sustained and progressive nature of the inflammation in CP.

It is conceivable that therapeutic approaches aiming to resolve mucoprotein obstruction can break the cycle of irreversible progression. Animal models suggest that therapeutic interventions at early-stage disease could have the greatest impact on progression and prognosis (Vonlaufen et al., 2011). However, the clinical frameworks of early CP are not established as diagnosis mostly relies on end-stage pathology (Kleeff et al., 2017). On the other hand, recurrent acute pancreatitis (RAP) is clinically well-defined syndrome (acute pancreatitis attacks without radiological evidence of CP), which may progress into CP; therefore, it could be conceptually viewed as potential early CP. Future studies in this subgroup of patients will advance our understanding on human pathogenesis, progression, and offer the possibility to investigate early intervention strategies.

Decreased pancreatic fluid secretion in CP may be associated with CFTR dysfunction (Hegyi et al., 2016) and we speculate that pharmacological targeting would be beneficial in CP. Increased ductal bicarbonate secretion could improve the hydration and the rheological properties of mucus and facilitate wash-out from the ductal system. To date a number of small molecule CFTR modulators have been developed to rescue the basic defect in patients with CF. Although drug discovery programs were designed to restore specific CFTR mutations, these agents may also promote the function of wild-type CFTR. *In vitro* data demonstrated that ivacaftor, the first approved CFTR modulator, has the potential to counteract tobacco-induced CFTR dysfunction in COPD (Sloane et al., 2012). These promising results suggest that already available modulators may be effective for the restoration of CFTR function in CP. Mechanical approaches to resolve ductal plugging have been in clinical use. Extracorporeal shock wave lithotripsy (ESWL) can be applied in chronic calcifying pancreatitis for the clearance of large calcified stones, which has proved very effective in alleviating pain symptoms, and some authors even reported improvement of exocrine/endocrine function of the gland (Choi and Kim, 2006). However, calcifications may develop long after mucoprotein plugging, when irreversible damage has already occurred. A very interesting clinical study tested the effect of bromhexine-hydrochloride in a small cohort of non-abstinent alcoholic CP patients (Tsujimoto et al., 2005). Bromhexine is an oral over-the-counter drug used as a bronchial mucolytic (Zanasi et al., 2017). Bromhexine use for 6 months improved clinical symptoms in 8/12 patients and importantly, significantly increased fecal chymotrypsin, indicating exocrine improvement. Although the mechanism of action is unknown, this finding encourages further studies on muco-active agents in CP.

In the present paper, we introduce important aspects of mucus secretion defect in CP such as localization, timing, molecular identification of secreted mucins, but there are several limitations. First, increased luminal mucus was apparent by histological analysis in CP; however, the fixation and embedding process may not optimally preserve intraluminal material and therefore interpretation of these results should be taken with caution. Due to our limited access to human samples, direct examination of mucus content in the pancreatic juice was not possible, although this would also help to elucidate the potential role of mucus as a biomarker in CP. Second, the triggering factor for mucin overproduction remains elusive. Inflammatory cell infiltration, cytokine production, and oxidative stress in CP are likely candidates, on the other hand, association of *Muc6* overexpression with impaired ion transport would argue that noxious agents that inhibit CFTR function would also induce mucin hypersecretion. To test these possibilities development of a translational *in vitro* model system will be necessary that allows quantitative examination of electrolyte transport and mucus secretion changes. To achieve this goal recently introduced disease relevant human organoid cultures may be instrumental (Hohwieler et al., 2017). Third, our assays show a link between CFTR dysfunction and mucus dehydration but further confirmation by mechanistic investigations is needed to elucidate the direct role of CFTR-mediated bicarbonate transport on the regulation of mucus secretion, mucus rheology, and the development of ductal obstruction in the pancreas.

## Conclusion

We have demonstrated here that mucus accumulates in the small pancreatic ducts in CP, which is a combined result of mucin hypersecretion and decreased ductal flush-out mechanism. The observations presented here are consistent with defective mucus hydration associated with impaired epithelial ion transport, which presents important therapeutic implications for CP.

## Author Contributions

AB designed and performed the experiments, analyzed the data, and wrote the paper. ZB and BK gave the technical support with experimental pancreatitis model with advice of ZR. JM gave the technical support and conceptual advice. MS and J-PK conducted the MRCP analyses with advice of JM. JD, ZZ-S, and JS gave the technical support on mucus measurement studies. LT provided the tissue samples and advised on histology. MM contributed to the study design and edited the manuscript. PH conceived and designed the study and wrote the manuscript.

## Conflict of Interest Statement

The authors declare that the research was conducted in the absence of any commercial or financial relationships that could be construed as a potential conflict of interest.
