# Supplementary material for: Ductal Mucus Obstruction and Reduced Fluid Secretion Are Early Defects in Chronic Pancreatitis
Source: Front Physiol. 2018 May 29;9:632. doi: 10.3389/fphys.2018.00632 (PMC5987707; doi:10.3389/fphys.2018.00632)
Supplement: Supplementary file 1 [file Data_Sheet_1.PDF]

Supplementary figure 1. Image analysis pipeline of mucus measurements.

For epithelial mucus density and luminal mucus measurements (left panel) we used calibrated, white balanced images (A). Ducts were marked along the basal membrane and cropped, perimeter (basal lamina length) measured (B). By phase analysis color channels were split (C). Using the green channel intensity threshold for mucus was set (D). Epithelial/luminal total area and mucus area were measured (E). Additionally, ductal diameter was estimated of ductal cross-sections. Epithelial mucus density and luminal mucus percentage was calculated[1].

To measure mucus content of heterogenous luminal content consisting of proteinaceous (magenta) material and alcian-blue mucus (right panel) we introduced an additional phase analysis step to determine the overall area of luminal content in the lumen. Due to the magenta saturation of these luminal images we determined mucus area in red channel and luminal content area in the green channel. Proteinaceous area was calculated by subtracting the luminal mucus area from the luminal content area.

Supplementary figure 2. Intraluminal mucus content increases in small pancreatic ducts

Luminal mucus (A) and luminal proteinaceous content (B) measurement of the pancreatic ducts in control and CP human tissues. N=9-9 tissue samples N=15-71 ducts per group

Luminal mucus measurement of pancreatic ducts in control and 4-week cerulein-treated mice N=9-9 tissue samples N=4-116 ducts per group. ***$P<0.001$, *$P<0.05$

1 Weibel, E. R.: Morphometry of the Human Lung. Springer Verlag, Berlin-Göttingen-Heidelberg 1963; 151 S., 109 Abb., DM 36,- - Lorenz - 1966 - Biometrical Journal - Wiley Online Library. http://onlinelibrary.wiley.com/doi/10.1002/bimj.19660080155/abstract (accessed 6 Feb 2018).
