# Supplementary figures and images for: Ductal Mucus Obstruction and Reduced Fluid Secretion Are Early Defects in Chronic Pancreatitis
Source: Front Physiol. 2018 May 29;9:632. doi: 10.3389/fphys.2018.00632 (PMC5987707; doi:10.3389/fphys.2018.00632)

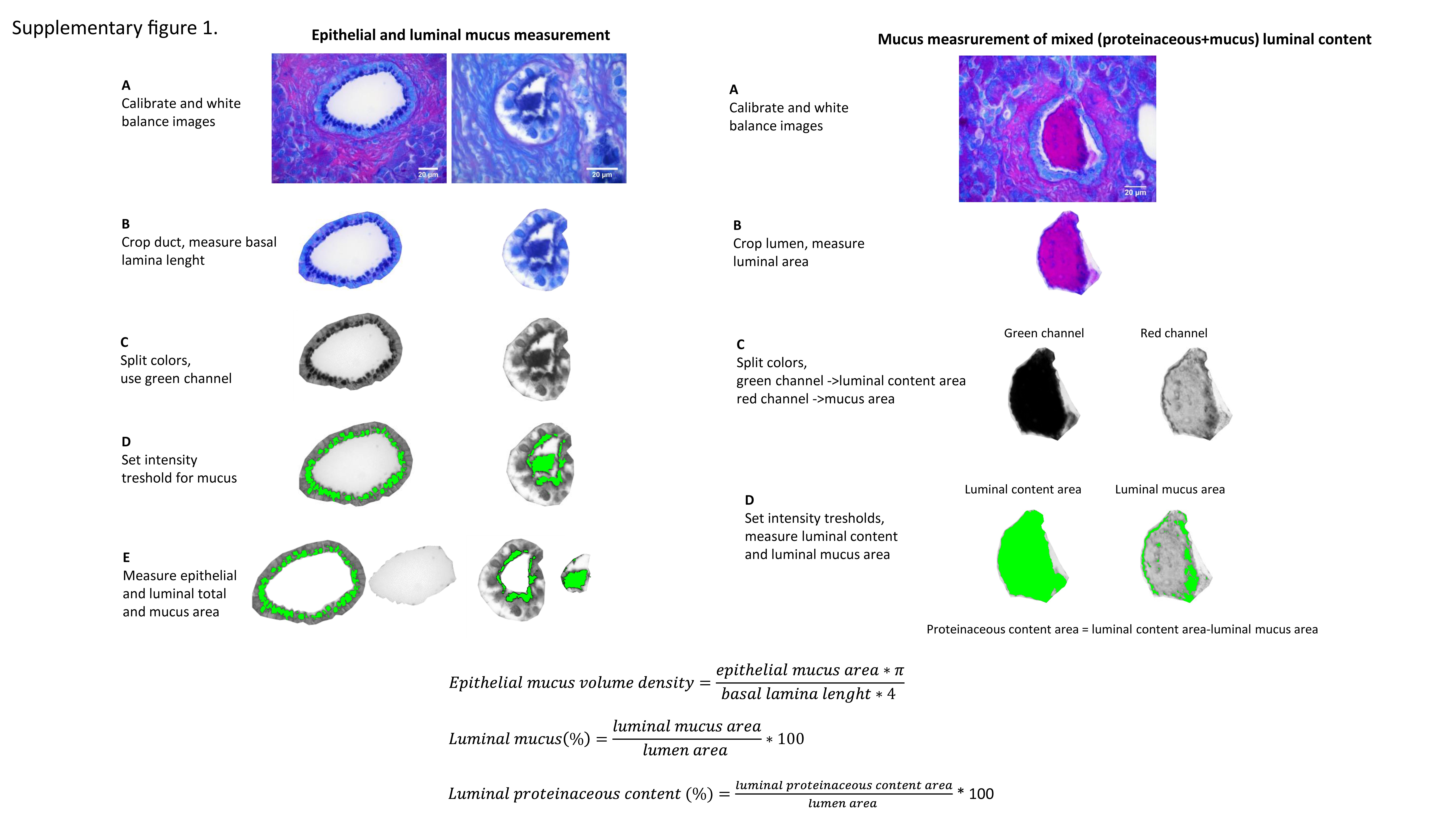

Supplement: Supplementary file 2 [file Image_1.jpeg]

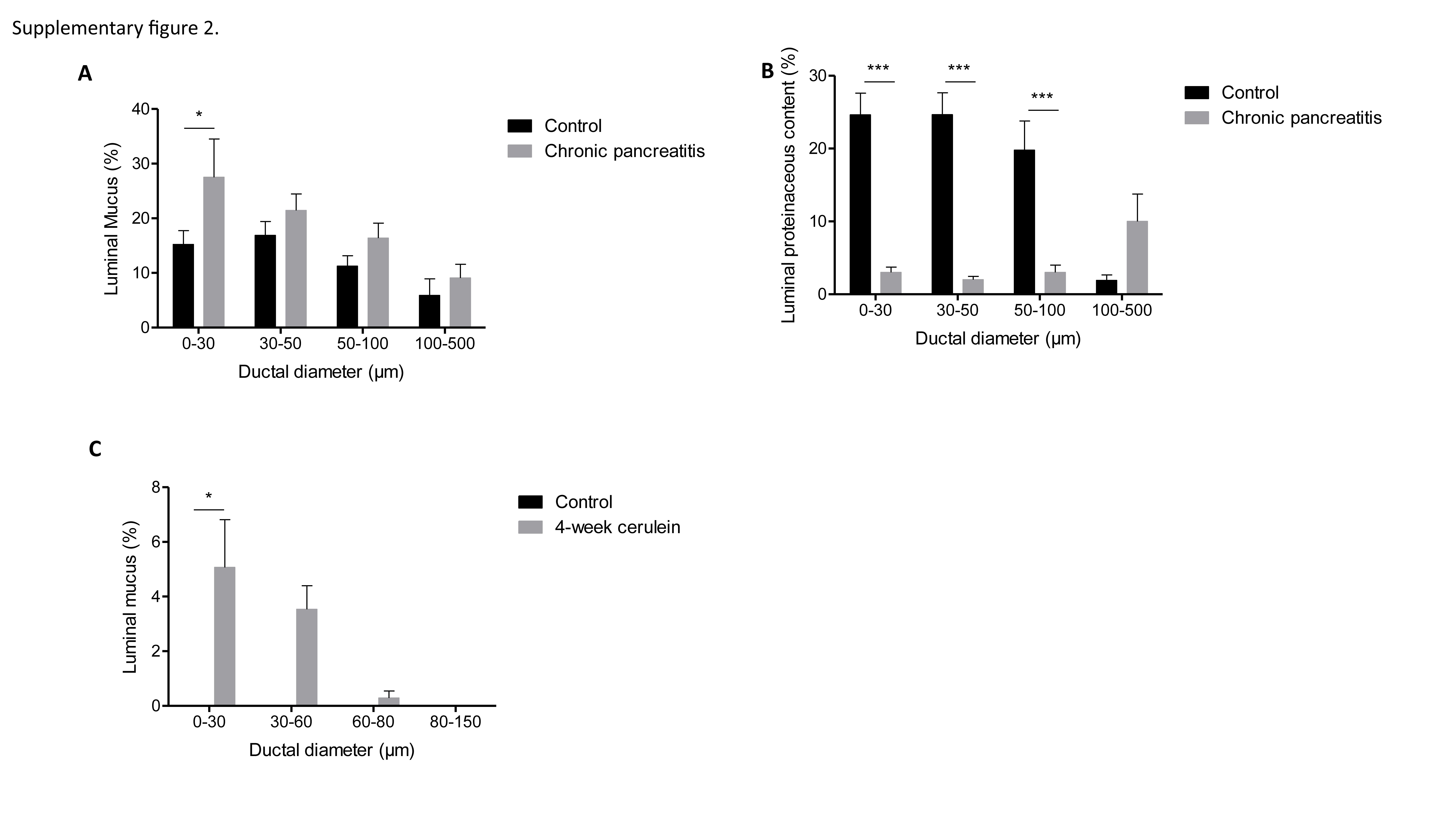

Supplement: Supplementary file 3 [file Image_2.jpeg]
